# Anti-Cholinergic Drug Burden Among Ambulatory Elderly Patients in a Nigerian Tertiary Healthcare Facility

**DOI:** 10.3389/fphar.2021.580152

**Published:** 2021-01-29

**Authors:** Joseph O. Fadare, Abimbola Margaret Obimakinde, Felix O. Aina, Ebisola J. Araromi, Theophilus Adekunle Adegbuyi, Oluwatoba E. Osasona, Tosin A. Agbesanwa

**Affiliations:** ^1^ Department of Pharmacology and Therapeutics, College of Medicine, Ekiti State University, Ado-Ekiti, Nigeria; ^2^ Department of Medicine, Ekiti State University Teaching Hospital, Ado-Ekiti, Nigeria; ^3^ Department of Community Medicine, Faculty of Clinical Sciences, College of Medicine, University of Ibadan, Ibadan, Nigeria; ^4^ Department of Family Medicine, University College Hospital Ibadan, Ibadan, Nigeria; ^5^ Department of Family Medicine, Ekiti State University Teaching Hospital, Ado-Ekiti, Nigeria

**Keywords:** anticholinergic agents, adverse drug reactions, elderly, ambulatory care, tertiary healthcare, Nigeria

## Abstract

**Background:** The use of drugs with anticholinergic effects among elderly patients is associated with adverse clinical outcomes. There is paucity of information about anticholinergic drug burden among Nigerian elderly population.

**Objectives:** To determine the anticholinergic drug burden among elderly Nigerian patients.

**Methods:** This was a retrospective cross-sectional study conducted among elderly patients (aged 65 and above) who visited the Family Medicine outpatients’ clinics of the Ekiti State University Teaching Hospital, Ado-Ekiti, Nigeria between July 1 and October 31, 2018. Information extracted from the case files included patient’s age, sex, diagnoses, and list of prescribed medications. Medicines with anticholinergic effects were identified and scored using the anticholinergic drug burden calculator (http://www.acbcalc.com).

**Results:** The medical records of 400 patients were analyzed with females accounting for 60.5% of the study population. The mean age of participants was 73 ± 7.4 years with only 28 (7%) of patients having more than two co-morbid conditions. Polypharmacy was identified in 152 (38%) of the patients while 147 (36.7%) had drugs with anticholinergic effects prescribed. The anticholinergic burden was high in 60 (15%) patients. Polypharmacy was significantly associated with having more than two diagnosed conditions and high anticholinergic burden (*p* value of < 0 .001 and 0.013 respectively). There was significant correlation between total number of prescribed drugs and count of diagnoses (r = 0.598; *p* < 0 .000) and between total number of prescribed drugs and number of drugs with anticholinergic effects (r = 0 .196; *p* < 0 .000).

**Conclusion:** The anticholinergic burden in this group of elderly Nigerian patients was low; majority (67%) had no exposure to drugs with anticholinergic effects with only 15% having high anticholinergic burden. Polypharmacy and multiple diagnosed conditions were positively associated with high anticholinergic burden. Based on the positive and significant correlations found in this study, a reduction in the number of prescribed medicines especially those with significant anticholinergic effects used for secondary indications may lessen the anticholinergic burden among the elderly.

## Introduction

Medication use among elderly patients is often associated with adverse drug reactions (Davies and O'Mahony, 2015). Studies have shown that elderly patients are more at risk of adverse drug reactions (ADRs) than younger patients because of prescription of multiple medicines on a background of multiple co-morbidities and physiological decline in major body systems (Lattanzio et al., 2012; Shah and Hajjar, 2012). Anticholinergic drugs are used in routine clinical practice for common conditions such as allergy, cough, dyspepsia, anxiety and insomnia (Nishtala et al., 2016; López-Álvarez et al., 2019). Categories of drugs with anticholinergic effects also include anti-psychotics, anti-depressants, anti-emetics and drugs used in the treatment of chronic obstructive airways disease and Parkinson’s disease (Gerretsen and Pollock, 2011; Lupu et al., 2017; Rogliani et al., 2018). Recognized adverse effects of anticholinergic drugs include dryness of the mouth, blurred vision, constipation, urinary retention, postural hypotension and confusion (Collamati et al., 2016). The use of drugs with anticholinergic effects by patients is associated with adverse clinical outcomes such as delirium, frailty, falls, and cognitive impairment (Cancelli et al., 2009; Cardwell et al., 2015; Naja et al., 2016; Green et al., 2019). Long-term use of drugs with anti-cholinergic effects has also been associated with increased frequency of re-admissions to the emergency unit, worsening morbidity and mortality (Lattanzio et al., 2018; Hwang et al., 2019). Elderly patients are more at risk of these adverse effects because of declining age-related physiologic changes in the blood-brain barrier, liver and kidneys and a reduction in the central cholinergic transmission (Cancelli et al., 2009; Andres et al., 2019; Koren et al., 2019). Drugs with anticholinergic effects are prominent in the list of potentially inappropriate medicines prescription for the elderly such as those listed in the Beers, STOPP and PRISCUS criteria (Holt et al., 2010; American Geriatrics Society, 2015; O'mahony, 2020). Several tools have been developed and validated to assess the burden of anticholinergic drugs among elderly patients. These include the Anticholinergic Risk Scale (ARS), Anticholinergic Cognitive Burden (ACB) Scale, Anticholinergic Drug Scale (ADS) and Drug Burden Index anticholinergic component (DBI-ACh) (Narayan et al., 2013; Salahudeen et al., 2015). These tools vary in terms of inclusion criteria and allocated scores for anticholinergic drugs, hence the non-comparability of studies using them (Welsh et al., 2018). The burden of anti-cholinergic drugs have been extensively studied among the elderly in Australia, Europe and the USA. (Magin et al., 2016; Niznik et al., 2017; Kiesel et al., 2018). This is understandable because of the relatively large proportion of the elderly and level of geriatric healthcare services provided in these countries. The population of the elderly continues to grow in Africa and as such we expect medicine-related problems to increase among them. Some research work has been conducted in Nigeria, South Africa, Togo and Ethiopia on medication related issues such as prescription of inappropriate medicines among the elderly (Fadare et al., 2013; Nwani and Isah, 2015; Saka et al., 2018; Tegegn et al., 2019; Gbeasor-Komlanvi et al., 2020). However, there is paucity of information about the use of drugs with anticholinergic effects and anticholinergic drug burden among elderly patients in Sub-Saharan Africa, especially in Nigeria. This is not surprising as the specialty of geriatric medicine is just being given necessary attention and recognition in Nigeria. This has some implications especially regarding rational use of medicines in the elderly. A recent study conducted among physicians in Nigeria showed that only about 30% of them had good knowledge about prescription of inappropriate medicines for the elderly. (Fadare et al., 2019). This study assessed the prevalence of prescription of drugs with anticholinergic effects among ambulatory elderly Nigerian patients, which is one of the possible ills of physician’s lack of knowledge about inappropriate medicines for the elderly. Additionally, we estimated the anticholinergic drug burden and factors that may influence it. The findings of this study will provide a template for the incorporation of a robust medication review mechanism for elderly Nigerian patients.

## Methods

### Study Setting

This was a retrospective cross-sectional study conducted among elderly patients (aged 65 and above) who visited the Family Medicine outpatients’ clinics of the Ekiti State University Teaching Hospital (EKSUTH), Ado-Ekiti, Nigeria between July 1 and October 31, 2018. This public tertiary healthcare facility is owned by the government of Ekiti State and serves the healthcare needs of the 3.2 million inhabitants of Ekiti State and clients from neighboring Osun, Ondo and Kogi states. The healthcare facility, which is affiliated with Ekiti State University College of Medicine, offers primary, secondary and tertiary level care through its team of consultants, resident doctors, pharmacists, nurses and other healthcare professionals. Majority of the patients access primary care at the Family Medicine clinics and are followed up there while those requiring the services of other specialists are referred appropriately. The clinic has a specialized geriatrics clinic to cater for the needs of elderly patients who account for a large proportion of patients presenting to the Family Medicine department.

### Study Population

Elderly patients aged 65 years and above attending the Family Medicine outpatients’ clinic of EKSUTH. Patients with missing relevant information in their case files were excluded from the study.

### Sampling

Systematic sampling technique was used for this study. According to the WHO methodology for drug utilization studies (World Health Organization, 1993), a minimum of 100 records per facility is required. Because the study was conducted in only one center, we used the records of 400 patients for this study in order to improve on its power and make the findings more robust.

### Sampling Technique

Based on our total sample of 400 patients, the medical records of 100 patients aged 65 years and above were extracted monthly for the duration of the study. Using the clinic attendance register, a research assistant (resident doctor) selected 25 patients file numbers every week during the duration of the study. Depending on the recorded daily patient population, a different interval was used to select five patients’ records daily throughout the study period. Inclusion of patients’ records stopped at the end of four weeks for months with more than four weeks. The selected case record files were later retrieved from the medical records department and necessary information extracted. In case of missing data in a patient’s records, this was replaced with another case record file following the protocol described above.

### Study Instrument

The data collection tool was developed for the purpose of this study based on our previous studies (Fadare et al., 2013; Fadare et al., 2015). Information extracted from the case files included patient’s age, sex, diagnoses (co-morbidities), and list of prescribed medications. Patients were classified as having low diagnosis count (with less two diagnosed conditions) and high diagnosis count (>2 diagnosed conditions. Drugs with anticholinergic effects were identified and scored using the anticholinergic drug burden calculator (Anticholinergic Burden, 2015). Classification of the severity of anticholinergic drug burden was done as follows: none (0), low (1–2) and severe (more than 2). For the purpose of this study, polypharmacy was defined as having more than four prescribed medicines. The severity of anticholinergic burden is classified as: none (score of 0), low (1–2) and severe (more than 2).

### Data Management

Obtained data was coded and analyzed using IBM SPSS Statistics for Windows, Version 23.0 (Armonk, NY: IBM Corp). Analysis using descriptive statistics was performed to obtain the general characteristics of the study participants. Chi square was used to determine the level of significance of groups of categorical variables such as age group, sex, diagnosis count (>2 or <2) those with or without polypharmacy, the presence or otherwise of prescribed drugs with anticholinergic effects and severity of anticholinergic burden. *p* values < 0.05 were considered significant. Correlation analysis was conducted to explain the relationships between total number of prescribed drugs, number of co-morbid conditions and number of drugs and anticholinergic activity.

### Ethical Considerations

Ethical clearance for the study was obtained from the Research Ethics Committee of the Ekiti State University Teaching Hospital before the commencement of the study. The confidentiality of patient information was protected through non-usage of patients’ identifiers on the data collection form. The collected data was also stored in a passworded computer accessible only to the principal investigator (lead author).

## Results

### Demographics and General Characteristics of Patients

**FIGURE 1:**
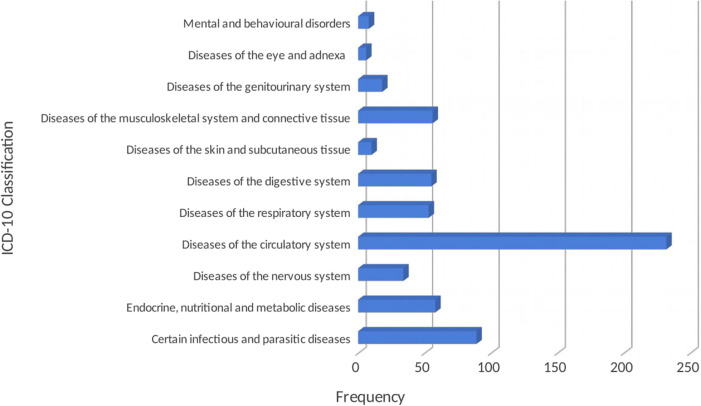
Classification of the diagnoses according to the ICD-10.

Information from the medical records of 400 patients was extracted and analyzed. The mean age of participants was 73.0 ± 7.4 years with female (60.5%) preponderance. Majority 218) of patients were diagnosed with one medical condition while 38.5%, 6 and 0.8% had two, three and four different diagnoses respectively. Hypertension (55.8%), malaria (20.3%), diabetes mellitus (14.3%) and osteoarthritis (12.3%) were the most common medical conditions among our cohort. Figure 1 shows classification of the diagnoses according to the ICD-10.

### Analysis of Prescribed Medicines

The mean number of prescribed medicines was 4.1 ± 1.5. Polypharmacy was identified in 38% of the patients while 37.0% had drugs with anticholinergic effects prescribed. Table 1 shows distribution of patients according to age, sex, count of diagnoses, number of prescribed drugs with anticholinergic effects, and anticholinergic burden. The means of total prescribed drugs for the stated categories are highlighted in Table 1.

**TABLE 1:** Socio-demographic details, classes of co-morbidities and anticholinergic burden with corresponding mean number of prescribed medicines.

Variable	Frequency (%)	Mean number of prescribed medicines	Comparison of means *p* Value
**Sex**			
Male	158 (39.5)	4.0 (1.5)	0.091
Female	242 (60.5)	4.2 (1.5)	
**Age group**			
65–74 years	243 (60.8)	4.1 (1.5)	0.167
75 and above	157 (39.2)	4.3 (1.5)	
**Diagnosis count**			
Low (1–2)	373 (93.3)	4.0 (1.4)	<0.0001^a^
High (>2)	27 (6.7)	6.4 (1.2)	
**Classification by anticholinergic burden score**			
No burden (ABS = 0)	253 (63.3)	3.8 (1.4)	0.001^a^
Low burden (ABS 1–2)	87 (21.7)	4.3 (1.3)	
High burden (3 and above)	60 (15.0)	4.8 (1.5)	
**Presence of drugs with anticholinergic effects in prescription**			
None	253 (63.3)	4.0 (1.4)	0.001^a^
Present	147 (36.7)	4.5 (1.5)	
**Polypharmacy**			
No	248 (62)		
Yes	152 (38)		
**Number prescribed drugs with anticholinergic effects**			
0	253 (63.0)		
1	117 (29.4)		
2	27 (6.8)		
3	3 (0.8)		

^a^ Indicates statistical significance.

^b^ Polypharmacy is defined as having more than four medications prescribed.

**FIGURE 2:**
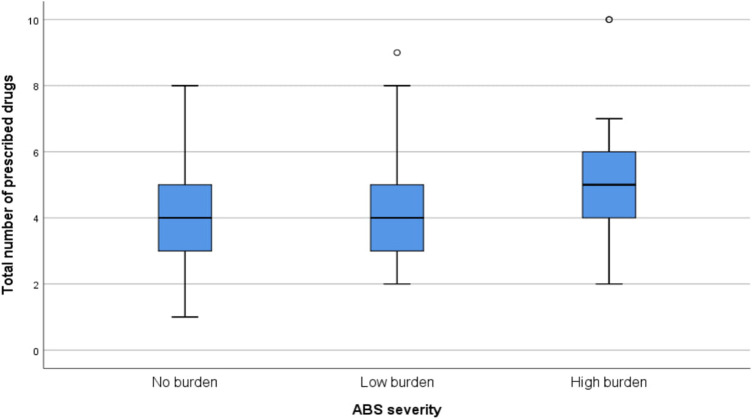
Relationship between number of prescribed medicines and severity of anticholinergic burden.

**FIGURE 3:**
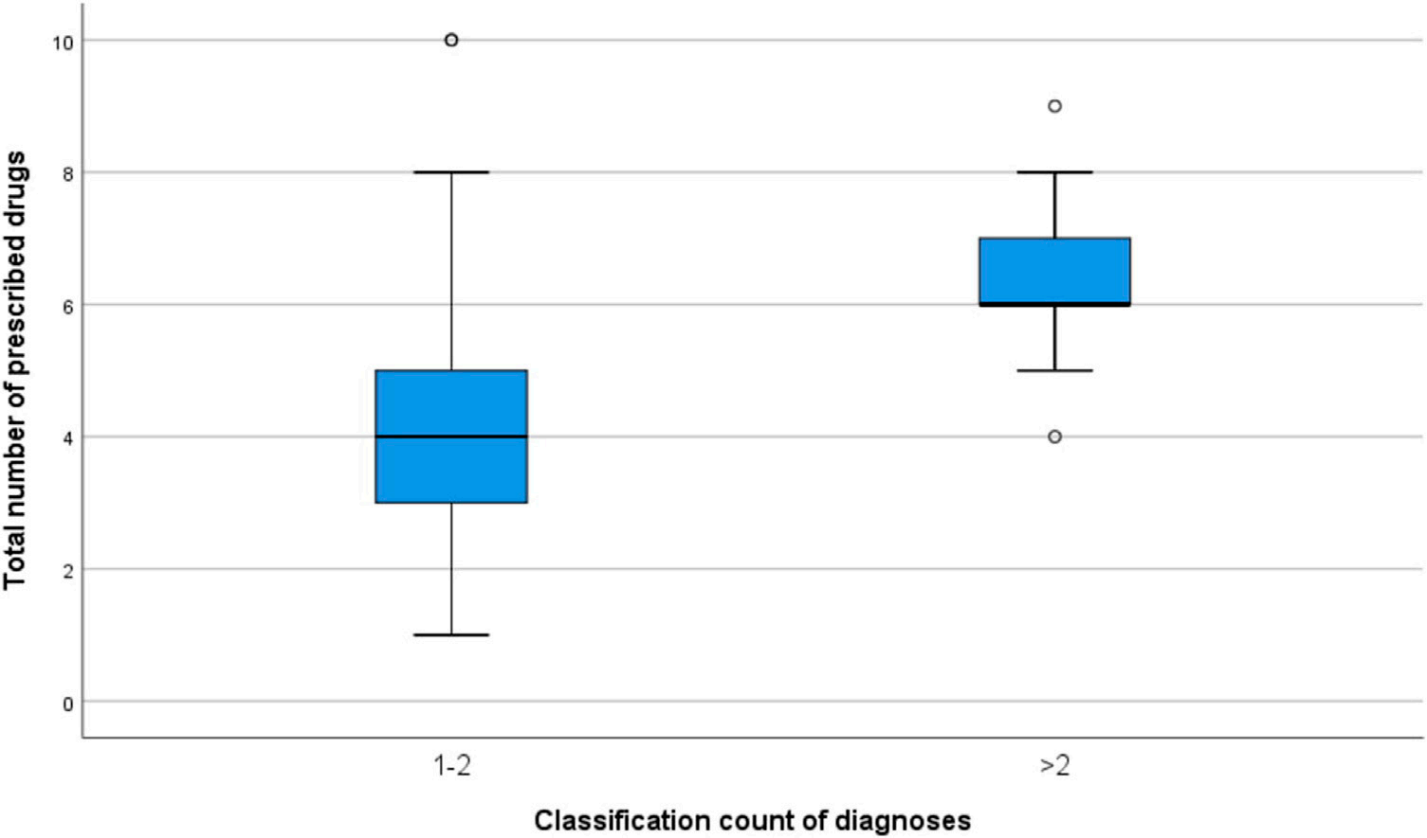
Relationship between the number of prescribed medicines and classification of diagnoses count.

Univariate analysis of the categories: number of prescribed drugs, count of diagnoses and severity of anticholinergic burden showed statistically significant associations (*p* values of <0.0001 and 0.042 respectively). The details of the univariate analyses are shown in Table 2. The relationship between number of prescribed drugs and severity of anticholinergic burden is shown in Figure 2 while that between the number of prescribed drugs and count of diagnoses is highlighted in Figure 3.

**TABLE 2:** Cross-tabulation of variable categories.

Variable	No polypharmacy	With polypharmacy	*p* value	No anticholinergic drugs	With anticholinergic drugs	*p* value
**Sex**						
Male	103 (25.8)	55 (13.8)	0.294	102 (25.5)	56 (14.0)	0.673
Female	145 (36.2)	97 (24.2)		151 (37.8)	91 (22.7)	
**Age group**						
65–74 years	158 (39.5)	85 (21.3)	0.140	159 (39.8)	84 (21.0)	0.289
75 and above	90 (22.5)	67 (16.7)		94 (23.5)	63 (15.7)	
**Diagnosis count**						
Low (1–2)	236 (59.0)	137 (34.3)	0.000^a^	239 (59.8)	134 (33.5)	0.217
High (>2)	0	27 (6.7)		14 (3.5)	13 (3.2)	
**Classification by anticholinergic burden score**						
No burden (ABS = 0)	164 (41.0)	89 (22.2)	0.042^a^	253 (63.3)	—	—
Low burden (ABS 1–2)	57 (14.3)	30 (7.5)		0	89 (22.2)	
High burden (3 and above)	27 (6.8)	33 (8.2)		0	58 (14.5)	

^a^ Indicates statistical significance.

^b^ Please add up all the cells in each segment of the table (i.e., classification by anticholinergic burden by polypharmacy/no polypharmacy to obtain 100%.

There was significant correlation between total number of prescribed drugs and count of diagnoses (r = 0.598; *p* < 0.000) and also between total number of prescribed drugs and number of drugs with anticholinergic effects (r = 0.196; *p* < 0.000).

According to their prevalence, drugs with anticholinergic effects identified in this study include amitriptyline, chlorpheniramine, diphenhydramine (a major component of cough syrups), loratadine, orphenadrine, tizanidine, loperamide, tramadol, cimetidine, trihexylphenidyl, carbamazepine and the centrally acting anti-hypertensives alpha-methyldopa and doxazosin. Other commonly prescribed medications identified in this study with relatively weaker anticholinergic effects according to the anticholinergic burden calculator include prednisolone, nifedipine, diazepam, furosemide, atenolol and hydrochlorothiazide.

## Discussion

This study assessed the prevalence of prescription of drugs with anticholinergic effects and anticholinergic burden among ambulatory elderly patients attending the Family Medicine outpatients’ clinic of EKSUTH, Ado-Ekiti, Nigeria. Approximately a third of patients had exposure to drugs with anticholinergic effects with 15% having a high anticholinergic burden.

### Analysis of Prescribed Medicines

The mean number of prescribed drugs in this study was 4.1, which is close to the mean range of 3.7–3.8 reported by studies conducted in Nigeria, Portugal and *Argentina* (Fadare et al., 2013; Pereira et al., 2017a; Chiapella et al., 2018). However, a slightly higher mean number of prescribed drugs with a range of 5.6–6.3 was reported in studies from Slovenia, Jordan, and Kuwait(Gorup and Rifel, 2018; Al-Azayzih et al., 2019; Awad and Hanna, 2019).

A plausible explanation for the aforementioned variation, may be the nature and number of co-morbid conditions, variation in age, availability of health insurance and peculiar medico-legal environment which may readily support higher number of medication prescription. Elderly patients with higher diagnosis count significantly had more drugs prescribed to them compared to those with less. This observation is in keeping with reports from several other studies where the presence of multiple co-morbidities in the elderly patients was a factor associated with higher number of prescribed medicines (Slabaugh et al., 2010; Gbeasor-Komlanvi et al., 2020). Elderly patients with higher number of prescribed drugs were more likely to have drugs with anticholinergic effects prescribed resulting in a higher anticholinergic burden, as revealed by results of this study. This finding has also been confirmed by many other studies were the risk of anticholinergic burden is associated with higher number of prescribed medicines in elderly patients (Nishtala et al., 2014; Sevilla-Sánchez et al., 2018).

Polypharmacy is a recognized risk factor for adverse drug reactions with consequences such as worsening morbidity, mortality and increasing healthcare costs. Polypharmacy was present in more than a third of our study participants; this is similar to a prevalence of 32% obtained in a Brazilian study (Pereira et al., 2017b). Contrarily, a study conducted among the elderly in Togo reported 22% prevalence of polypharmacy, while a pan-European study reported prevalence in range of 26.3–39.9% (Midão et al., 2018; Gbeasor-Komlanvi et al., 2020).

### Anticholinergic Burden

This study revealed that one hundred and forty-seven (36.7%) elderly patients had exposure to at least one drug with anticholinergic effects. It could be argued that our finding falls within reports from studies conducted among community-dwelling elderly patients in Slovenia, China and USA with prevalence of 12.5, 23.8, 27.1% and was significantly lower than 82% found in a Spanish study (Ness et al., 2006; Gorup and Rifel, 2018; Sevilla-Sánchez et al., 2018; Zhang et al., 2019). The variations in these studies may be related to the pattern of prescriptions which of course will be peculiar to the disease epidemiology in these different parts of the world. In particular, the higher prevalence of prescription and use of drugs with anticholinergic effects may be due to different diagnosed conditions such as Alzheimer’s disease and other degenerative conditions not commonly diagnosed in Nigeria. Majority (63.2%) of patients had no exposure to drugs with anticholinergic effects; this may have contributed to the reported low anticholinergic burden in this study. The anticholinergic burden was classified as none, low and high in this study and using this categorization we found 22 and 15% prevalence for low and high anticholinergic burden respectively. On the contrary, Teri-West *et al* in their study conducted among community-dwelling elderly in the USA reported 47.8% of the elderly persons with high anticholinergic burden consequent to their prescribed medications(West et al., 2013). Generally, high anticholinergic burden has also been reported in patients being managed for urological, psychiatric and neurological disorders (Crispo et al., 2016; Lupu et al., 2017; Yoshida et al., 2018). Therefore, it is not surprising that our study revealed low anticholinergic burden, since our study population were family practice patients, majority of whom are receiving only primary care with few being co-managed by other specialists. Likewise, elderly patients with long-standing psychiatric, neurological and urological problems who are likely to have high anticholinergic burden were not many in this study as they would have presented directly or referred after initial presentation to respective specialist clinics. Additionally, the relatively low anticholinergic burden found in our study may be due to the fact that some of the anticholinergic medicines used commonly in Europe and the USA are not available in the Nigerian pharmaceutical market as they are not listed in the latest version of the National Essential Medicines List (Federal Ministry of Health and Nigeria, 2016).

The most commonly used drugs with anticholinergic effects in this study were amitriptyline, diphenhydramine and chlorpheniramine. Tramadol, zopiclone and zolpidem were mostly implicated in a study in the United Kingdom while the antidepressants amitriptyline, nortriptyline and paroxetine were reported in a study from New Zealand (Narayan et al., 2013; Byrne et al., 2018). These different patterns of prescriptions may be due to variation in disease presentations in the aforementioned countries and the composition of their national essential medicines lists.

### Reported Diagnosed Conditions

Hypertension, diabetes and osteoarthritis were the most common chronic diseases diagnosed among patients in this study, similar to findings from other Nigerian studies conducted at primary care level (Fadare et al., 2013; Cadmus et al., 2017). This seems to suggest that some of the prescribed medicines with anticholinergic effects in our study were prescribed for their secondary indications. For example, the tricyclic antidepressant, amitriptyline is also used in the management of peripheral neuropathy caused by diabetes (Cohen et al., 2015; Liampas et al., 2020); this may explain its relatively common use among this group of patients. The use of antihistamines such as chlorpheniramine and diphenhydramine may be explained by respiratory tract infections diagnosed among the patients. This raises a question on the rationality of the routine use of antihistamines for the treatment of respiratory tract infections among the elderly. Also, medicines such as cimetidine, nifedipine and prednisolone used in the management of common conditions among this group of patients had low anticholinergic burden score. This brings to the fore the need for prescribers, especially those caring for the elderly, to constantly update their knowledge in the area of appropriate prescribing for the elderly.

#### Study Limitations and Strengths

This study was conducted in a single tertiary healthcare facility; hence, its findings may not be nationally representative and also may not reflect what is obtainable in other levels of care and in private hospitals. Most elderly patients in Nigeria patronize government-owned healthcare facilities because of perceived lower costs hence we believe that this study captured a sizable proportion of elderly patients who presented at the teaching hospital during the duration of the study. Limitations associated with retrospective studies such as missing information, inability to substantiate data and selection bias may have some implications on the study outcome. This possibility was addressed through the classification of disease conditions according to systems (ICD-10 classification) and appropriate sampling technique used in the study. Another factor that may affect the interpretation of our results is seasonal variation in presentation of diseases; the study was conducted during the rainy season with a preponderance of respiratory tract infections among the population and as such there may be high reported rate of prescription of medicines with anticholinergic properties (antihistamines).

The relatively large sample size of 400 can be described as the major strength of this study. Also, the fact that this study is novel in Nigeria and addresses a topical issue that is often neglected in Sub-Saharan Africa further strengthens the study. The study provides preliminary data for further studies on anticholinergic burden among the Nigerian elderly population.

## Conclusion

The anticholinergic burden in this group of elderly Nigerian patients was low; majority (67%) had no exposure to anticholinergic medications with only 15% having high anticholinergic burden. Polypharmacy and multiple diagnosed conditions were positively associated with high anticholinergic burden. The use of medicines with anticholinergic effects for secondary indications and their non-rational use was found to have contributed significantly to the anticholinergic burden reported in this study. Based on the above, a reduction in the number of prescribed medicines especially those with significant anticholinergic effect used for secondary indications is recommended. Also, non-rational prescription of medicines with anticholinergic activity for elderly patients should be discouraged.

## Data Availability Statement

The raw data supporting the conclusions of this article will be made available by the authors, without undue reservation. Requests to access these datasets should be directed to joseph.fadare@eksu.edu.ng.

## Ethics Statement

The studies involving human participants were reviewed and approved by Research Ethics Committee, Ekiti State University Teaching Hospital, Ado-Ekiti, Nigeria. Written informed consent for participation was not required for this study in accordance with the national legislation and the institutional requirements.

## Author Contributions

JF, Conceptualization of the study, data analysis and drafted the manuscript; AO, Literature review, review of manuscript for intellectual content; FA, Literature review, data acquisition and analysis, review of manuscript for intellectual content; EA, Data acquisition and analysis, review of manuscript for intellectual content; OO, Literature review, review of manuscript for intellectual content; TAd, Data acquisition and analysis, review of manuscript for intellectual content; TAg, Data acquisition and analysis, review of manuscript for intellectual content.

## Conflict of Interest

The authors declare that the research was conducted in the absence of any commercial or financial relationships that could be construed as a potential conflict of interest.
